# A Multi-Omics Analysis of a Mitophagy-Related Signature in Pan-Cancer

**DOI:** 10.3390/ijms26020448

**Published:** 2025-01-07

**Authors:** Nora Agir, Ilias Georgakopoulos-Soares, Apostolos Zaravinos

**Affiliations:** 1 Department of Life Sciences, School of Sciences, European University Cyprus, Nicosia 1516, Cyprus; na202005@students.euc.ac.cy; 2 Cancer Genetics, Genomics and Systems Biology Laboratory, Basic and Translational Cancer Research Center (BTCRC), Nicosia 1516, Cyprus; 3 Institute for Personalized Medicine, Department of Biochemistry and Molecular Biology, The Pennsylvania State University College of Medicine, Hershey, PA 17033, USA; izg5139@psu.edu

**Keywords:** mitophagy, *MAP1LC3*, *SRC*, *OPTN*, *PINK1*, *PARK2*, *PRKN*, *BECN1*, *BNIP3L*, drug sensitivity, drug resistance, multi-omics, pan-cancer

## Abstract

Mitophagy, an essential process within cellular autophagy, has a critical role in regulating key cellular functions such as reproduction, metabolism, and apoptosis. Its involvement in tumor development is complex and influenced by the cellular environment. Here, we conduct a comprehensive analysis of a mitophagy-related gene signature, composed of *PRKN*, *PINK1*, *MAP1LC3A*, *SRC*, *BNIP3L*, *BECN1*, and *OPTN*, across various cancer types, revealing significant differential expression patterns associated with molecular subtypes, stages, and patient outcomes. Pathway analysis revealed a complex interplay between the expression of the signature and potential effects on the activity of various cancer-related pathways in pan-cancer. Immune infiltration analysis linked the mitophagy signature with certain immune cell types, particularly *OPTN* with immune infiltration in melanoma. Methylation patterns correlated with gene expression and immune infiltration. Mutation analysis also showed frequent alterations in *PRKN* (34%), *OPTN* (21%), *PINK1* (28%), and *SRC* (15%), with implications for the tumor microenvironment. We also found various correlations between the expression of the mitophagy-related genes and sensitivity in different drugs, suggesting that targeting this signature could improve therapy efficacy. Overall, our findings underscore the importance of mitophagy in cancer biology and drug resistance, as well as its potential for informing treatment strategies.

## 1. Introduction

**Figure 1:**
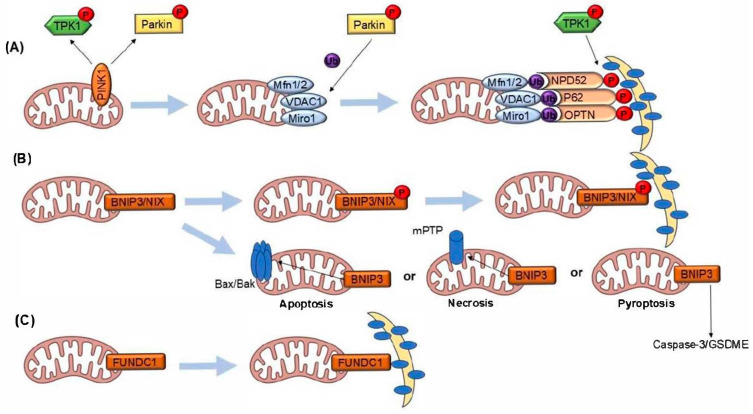
**Mechanisms of mitophagy and associated mitochondrial pathways.** (**A**) PINK1/Parkin-mediated mitophagy: Under mitochondrial stress, PINK1 accumulates on the outer mitochondrial membrane and recruits the E3 ubiquitin ligase Parkin. Parkin ubiquitinates mitochondrial outer membrane proteins such as Mfn1/2, VDAC1, and Miro1. These ubiquitinated proteins are subsequently recognized by autophagy receptors (NDP52, p62, and OPTN), which facilitate the recruitment of the autophagy machinery, leading to mitochondrial degradation. This pathway is regulated by phosphorylation events mediated by TPK1. (**B**) BNIP3/NIX-mediated mitophagy and cell death pathways: BNIP3 and its homolog NIX, both regulated through phosphorylation, can promote mitophagy by directly interacting with autophagy machinery. Alternatively, BNIP3 induces mitochondrial permeabilization through Bax/Bak activation, leading to the opening of the mitochondrial permeability transition pore (mPTP) and dissociation of the COX1-UCP3 complex. This can trigger apoptosis, necrosis, or pyroptosis. In pyroptosis, BNIP3 activation is linked to Caspase-3/GSDME activity. (**C**) FUNDC1-mediated mitophagy: FUNDC1, a mitochondrial outer membrane protein, undergoes phosphorylation-dependent regulation to mediate mitophagy by interacting with autophagic components, enabling the selective degradation of damaged mitochondria.

Mitophagy is a selective form of autophagy responsible for removing damaged or dysfunctional mitochondria. It plays a critical role in maintaining cellular health by preventing the accumulation of reactive oxygen species (ROS) and promoting mitochondrial quality control [1,2]. First described in yeast [3,4], mitophagy was later found to be mediated in mammalian cells through key pathways involving PINK1 and PRKN. Unlike general autophagy, which targets diverse cellular components, mitophagy specifically eliminates mitochondria. In contrast to apoptosis, mitophagy is a survival mechanism that sustains cellular function under stress. Causes of defective mitochondria include oxidative damage, mutations in mitochondrial DNA, and disruptions in mitochondrial dynamics. There are several mechanisms of mitophagy and associated mitochondrial pathways, as described in Figure 1.

A mitophagy-related gene signature, including the microtubule-associated protein 1 light chain 3 alpha (*MAP1LC3A* or *LC3*), PTEN-induced putative kinase 1 (*PINK1*), Parkin RBR E3 ubiquitin protein ligase (*PRKN or PARK2*), SRC proto-oncogene, non-receptor tyrosine kinase (*SRC*), optineurin (*OPTN*), BCL2/adenovirus E1B-interacting protein 3-like (*BNIP3L*), and Beclin 1 (*BECN1*), offers a comprehensive framework for understanding the mechanisms underlying mitochondrial quality control. MAP1A is a microtubule-associated protein which mediates the physical interactions between microtubules and components of the cytoskeleton. It consists of a heavy chain subunit and multiple light chain (LC) subunits. The protein encoded by MAP1A is one of the light chain subunits. LC3 is central to autophagosome formation and acts as a scaffold for replication [5,6]. Diseases associated with *MAP1LC3A* include neurodegeneration with brain iron accumulation and retinitis pigmentosa–deafness syndrome [7,8].

PINK1 and PRKN mediate the recognition and ubiquitination of damaged mitochondria [9]. The PINK1/PRKN pathway is closely associated with Parkinson’s disease and influences various cell processes in cancer cells [10]. When the inner mitochondrial membrane (IMM) is depolarized, PINK1 accumulates in the outer mitochondrial membrane (OMM) and phosphorylates Parkin, facilitating its recruitment to the OMM [11]. PINK1 plays a role in regulating various physiological and pathological processes in cancer cells, such as cytoplasmic homeostasis, cell survival, and cell death. Depending on the context, PINK1 can function as either a tumor promoter or suppressor [10].

SRC and OPTN facilitate the recruitment of autophagic machinery to defective mitochondria [12]. SRC plays a critical role in connecting damaged mitochondria with autophagy machinery, initiating their breakdown. Increased SRC activity is linked with mitochondrial damage and renal failure [13,14].

Disruptions in OPTN can interfere with mitophagy, potentially leading to the build-up of dysfunctional mitochondria and contributing to diseases such as amyotrophic lateral sclerosis (ALS) and glaucoma [15]. Optineurin facilitates the selective removal of damaged mitochondria through autophagosomes, specialized structures responsible for targeting and disposing of cellular components [16]. This process involves optineurin connecting with impaired mitochondria and guiding them to autophagosomes for degradation [17].

BNIP3L encodes for a mitophagy receptor, critical for the elimination of dysfunctional mitochondria [18]. BNIP3L has an increased activity in hypoxic conditions, interacting with autophagosomes through the protein LC3. In addition, it supports cancer stem cells and enhances metabolic plasticity. In normal conditions, BNIP3L help cells adapt in low-oxygen environments. Therefore, it plays a dual role in cancer as a tumor suppressor and as a tumor enhancer due to its survival aiding properties [19].

ΒECN1 is a core component of the class III phosphatidylinositol 3-kinase (PI3K-III) complex, which plays an important role in membrane trafficking and restructuring involved in autophagy [20]. Being a key component of the autophagosome, it contributes to drug resistance and allows cancer to evade chemotherapy. There is a strong connection between BECN1-mediated autophagy and the hypoxic conditions commonly present in the tumor microenvironment (TME), which in turn contributes to resistance against broad-spectrum anticancer drugs, such as bevacizumab [21,22].

Emerging evidence indicates that dysregulated mitophagy is closely associated with cancer, where mitochondrial dysfunction contributes to tumorigenesis, cancer cell survival, and resistance to therapy [23,24,25]. For instance, impaired mitophagy can lead to the accumulation of damaged mitochondria, resulting in increased reactive oxygen species (ROS) production and genomic instability, both of which are key drivers of cancer progression [26]. Furthermore, cancer cells often exploit mitophagy to survive under metabolic stress and adapt to hypoxic conditions within the TME [27]. Additionally, mitophagy has been implicated in the resistance of cancer cells to chemotherapy and radiotherapy, as the removal of damaged mitochondria helps in mitigating cell death [28,29].

The dual role of mitophagy in cancer is influenced by the TME and cancer stage. In early-stage tumors, mitophagy can act as a tumor suppressor by preventing the accumulation of damaged mitochondria, which produce reactive oxygen species (ROS) and promote genomic instability. Conversely, in advanced tumors, mitophagy can enhance survival under metabolic stress or hypoxia, allowing cancer cells to adapt to adverse conditions [30,31]. This dichotomy underscores the importance of context when targeting mitophagy in cancer therapy. Despite the growing understanding of mitophagy in cancer, significant gaps still remain in the literature. One major gap is the differential expression of this mitophagy-related gene signature across various cancer types and its impact on tumor biology and patient prognosis. While some studies have already explored this [32,33,34,35,36], a comprehensive pan-cancer analysis is lacking. Another critical area of investigation is the infiltration of immune cells in tumors with different levels of expression of this signature. The interplay between mitophagy and the TME could reveal new insights for immunotherapy [37]. Additionally, the deciphering of the patterns of mutations and methylation in mitophagy-related genes, and how they influence mitophagy and cancer progression, still remains underexplored. Also, understanding the sensitivity of different cancers with varying levels of this mitophagy-related gene signature to different drugs, as well as deciphering a potential link between mitophagy and drug resistance, could help us re-evaluate different personalized treatment strategies.

The investigation of this gene signature should provide valuable insights into the molecular pathways of mitophagy in the context of cancer and highlight potential therapeutic targets for modulating mitochondrial health and improving cancer treatment outcomes. For example, targeting PINK1 and PRKN to inhibit mitophagy could sensitize cancer cells to treatment by promoting the accumulation of dysfunctional mitochondria and enhancing oxidative stress [38,39]. Similarly, the modulation of the activity of MAP1LC3A (LC3) and OPTN could influence autophagosome formation and selective degradation of mitochondria, thereby affecting cancer cell survival [40].

In this study, we comprehensively explored the contribution of these genes to mitophagy and their implication in cancer biology, therapy, and drug resistance. We delved into the molecular mechanisms by which *LC3*, *PINK1*, *PRKN*, *SRC*, *BNIP3L*, *BECN1*, and *OPTN* regulate mitophagy, cancer progression and drug sensitivity or resistance.

## 2. Results

### 2.1. Differential Expression of the Mitophagy Signature in Pan-Cancer

We observed significant differential expression patterns of the mitophagy-related signature across various cancer types. Specifically, in breast cancer (BRCA) and head and neck squamous cell carcinoma (HNSC), *PRKN*, *PINK1*, and *MAP1LC3A* were downregulated, while *SRC* and *BECN1* were upregulated. In prostate adenocarcinoma (PRAD), *OPTN*, *PRKN*, *BNIP3L*, *PINK1*, and *MAP1LC3A* were significantly downregulated. In addition, *PRKN* was markedly downregulated in lung adenocarcinoma (LUAD), colon adenocarcinoma (COAD), thyroid cancer (THCA), and lung squamous cell carcinoma (LUSC), respectively. The expression of *BNIP3L* was high in kidney clear cell carcinoma (KIRC), but low in BRCA and chromophobe kidney cancer (KICH). *SRC*, on the other hand, was significantly upregulated in BRCA, squamous cell lung cancer (LUSC) and thyroid cancer (THCA), whereas *BECN1* was significantly downregulated in KIRC (Figure 1A,B and Supplementary Table S1). The observed expression trends of the mitophagy-related signature, especially *PRKN*, *PINK1* and *SRC*, may suggest (but do not imply) causative biological roles of these genes in tumorigenesis.

**Figure 2:**
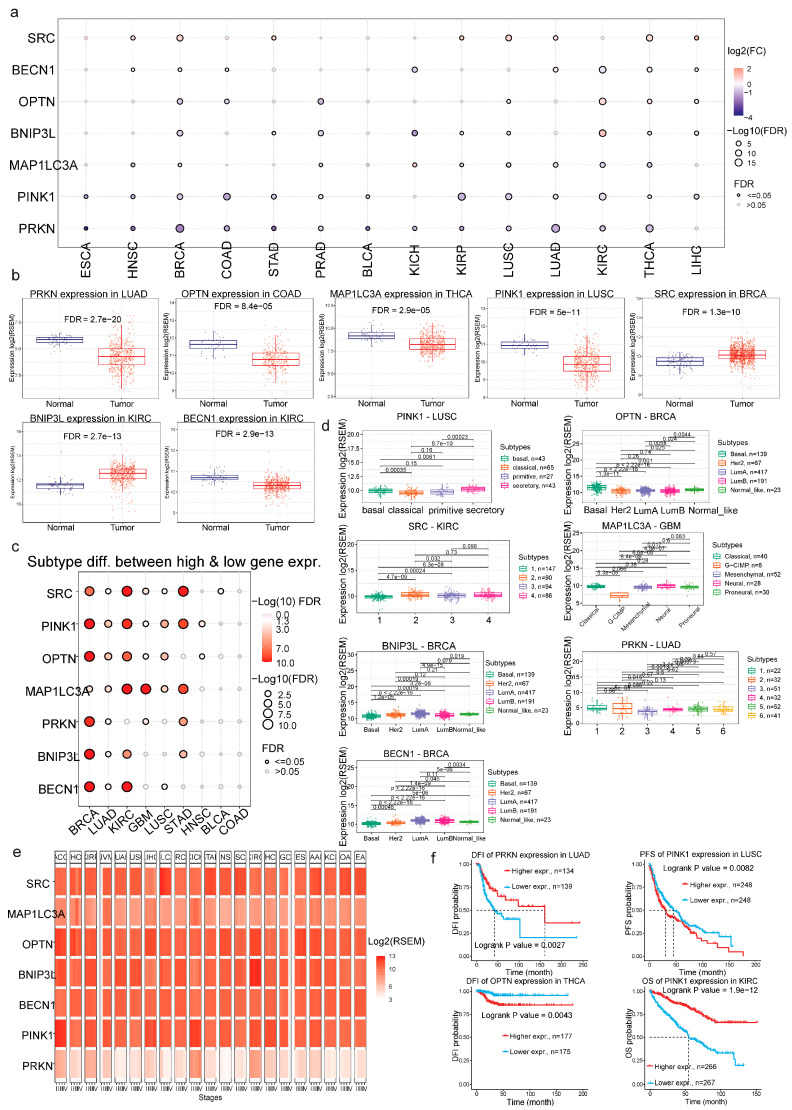
**Differential expression of the mitophagy-related signature in pan-cancer.** (**a**) The bubble plot illustrates the log_2_ fold change (FC) in the expression of the mitophagy-related genes across a spectrum of cancer types, with significance denoted by the false discovery rate (FDR) values, being represented by the color and size of the bubbles. Blue indicates downregulation, while red indicates upregulation of each gene in the tumor versus the normal tissues. The size of the circles is associated with FDR significance. Notably, *PRKN* and *PINK1* show substantial upregulation in lung adenocarcinoma (LUAD) and lung squamous cell carcinoma (LUSC), while *SRC* and *OPTN* are significantly elevated in breast cancer (BRCA) and colon adenocarcinoma (COAD), respectively. *BECN1* was significantly downregulated in KIRC, in contrast to *BNIP3L*, which was upregulated in the tumor. (**b**) Boxplots for selected cancer types, showcasing significant differences in gene expression between normal and tumor tissues. (**c**) Subtype-specific expression differences, emphasizing that certain genes exhibit distinct expression patterns within cancer subtypes, such as *SRC* in kidney renal clear cell carcinoma (KIRC) and *OPTN* in BRCA. Red large circles represent deregulation in mRNA expression that is statistically significant. (**d**) The boxplots depict expression differences (log_2_ RSEM) across molecular subtypes in different cancer types (*PINK1* in LUSC, *OPTN* in BRCA, *SRC* in KIRC, *MAP1LC3A* in GBM, *BNIP3L* and *BECN1* in BRCA, and *PRKN* in LUAD). (**e**) The heatmap shows a general trend of stable or decreased (in some cases like *SRC* in BLCA or *PINK1* and *OPTN* in ACC) expression for the mitophagy-related signature with advancing tumor stages. (**f**) The Kaplan–Meier curves show the survival rates in different types of cancer, according to gene expression. Higher expression of *PRKN* in LUAD and of *PINK1* in LUSC is associated with poorer prognosis, thereby underscoring the clinical relevance of these mitophagy-related genes in cancer progression and patient survival.

Subtype-specific investigations revealed that *SRC* and *PINK1* expression varied significantly among subtypes 1–4 in KIRC, stomach adenocarcinoma (STAD) and BRCA molecular subtypes (Basal, Her2, LumA, LumB, normal-like). *OPTN* expression also varied among BRCA, KIRC, LUAD, and LUSC subtypes. *MAP1LC3A* expression differed among classical, G-CIMP, mesenchymal, neuronal, and proneuronal subtypes in glioblastoma (GBM) but also among KIRC and STAD subtypes. *PRKN* expression differed among BRCA and STAD molecular subtypes. Likewise, *BECN1* and *BNIP3L* were differentially expressed in discrete subtypes of BRCA, KIRC and STAD (Figure 2c,d and Supplementary Table S2). Additionally, stage-wise analysis indicated a general trend of either decreasing or stable expression of the mitophagy signature with advancing stages in many cancer types (Figure 2e and Supplementary Table S3).

Importantly, survival analyses demonstrated that higher expression levels of *PRKN* and *PINK1* were associated with better patient outcomes in LUAD (DFI) and KIRC (OS), respectively. On the other hand, high expression levels of *PINK1* and *OPTN* correlated with worse patient outcomes in LUSC (PFS) and THCA (DFI), respectively (Figure 2f and Supplementary Table S4).

Altogether, the differences in the expression of the mitophagy-related signature between tumor and normal tissues, as well as across different molecular subtypes and cancer stages, supported by their correlation with patient survival and highlighted the potential of these genes as biomarkers for tumor prognosis.

### 2.2. Pathway Activity of the Mitophagy Signature in Pan-Cancer

**Figure 3:**
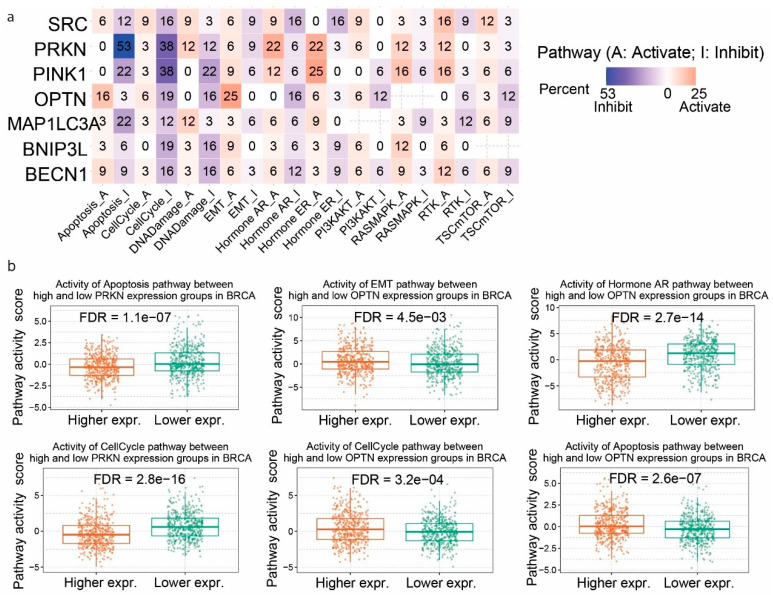
**Pathway activity associated with differential expression of mitophagy-related genes.** (**a**) Heatmap depicting the potential activation (A) or inhibitory (I) effects of the mRNA levels of the mitophagy-related gene signature on the activity of 10 cancer-related pathways in pan-cancer. The color scale indicates the percentage of pathway activation (red) or inhibition (blue). The percentages represent the frequency of gene association with pathway regulation in various types of cancer. (**b**) The boxplots compare the pathway activity scores (PAS) between high and low expression groups of *PRKN* and *OPTN* in BRCA. The FDR values indicate the significance of the differences in PAS found between the high and low expression groups.

We then explored the pathway activity associated with the differential expression of the mitophagy-related genes in pan-cancer. Ten cancer-related pathways were examined, each with a pair representing activation and inhibition. We found that higher *SRC* expression is significantly associated with activated RTK (receptor tyrosine kinase; 16%) and TSC/mTOR (tuberous sclerosis complex/mechanistic target of rapamycin; 12%) pathways in pan-cancer, indicating its potential role in promoting cell growth and survival. In addition, *SRC* upregulation was implicated at a lower rate in the activation of the cell cycle (9%), DNA damage (9%), hormone AR (hormone androgen receptor; 9%) and PI3K/AKT (phosphoinositide 3-kinase/protein kinase B; 9%) pathways. On the other hand, *SRC* displayed a potential inhibitory effect on the cell cycle (16%), hormone AR (16%), hormone ER (hormone estrogen receptor; 16%) and apoptosis (12%) pathways in pan-cancer (Figure 3a).

Likewise, the mRNA levels of *PRKN* exhibited a potential inhibitory effect on the activity of apoptosis (53%), the cell cycle (38%), and DNA damage (12%) pathways, but also demonstrated a potential activating effect on the activity of pathways like hormone AR (22%), hormone ER (22%), RTK (12%), and DNA damage (12%) in pan-cancer (Figure 3a). For example, the effects of higher pathway activity scores found in breast tumors expressing low levels of *PRKN* was quite evident (Figure 3b).

In addition, *PINK1* expression showed a potential inhibitory effect on the activity of the cell cycle (38%), apoptosis (22%) and DNA damage (22%) pathways in pan-cancer, further highlighting its potential role in inhibiting programmed cell death and contributing to tumor progression. It also showed potential activating effect on the activity of the hormone ER (25%), RAS/MAPK (RAS/mitogen-activated protein kinase; 16%) and hormone AR (12%) pathways (Figure 3a).

We also show that activated *OPTN* has a potential activating effect on the activity of EMT (25%) and Apoptosis (16%) pathways, as well as a potential inhibitory effect on the activity of the Cell cycle (19%), DNA damage (16%) hormone AR (16%) and PI3K/AKT (12%) in pan-cancer (Figure 3a). Across all tumors investigated, the most notable differences in the pathway activity scores between highly and lowly expressing groups, was shown in breast cancer (Figure 3b).

In addition, high mRNA levels of *MAP1LC3A* were shown to potentially activate the DNA damage response (12%) pathway, while potentially inhibit the activity of apoptosis (22%), the cell cycle (12%) and RTK (12%) pathways in pan-cancer (Figure 3a).

Furthermore, *BNIP3L* and *BECN1* expression potentially inhibited the cell cycle and DNA damage pathways (16–19%) in pan-cancer (Figure 3a).

Overall, our analysis revealed a complex interplay between the expression of the mitophagy-related gene signature and potential activating or inhibitory effects on the activity of various cancer-related pathways in pan-cancer.

### 2.3. Correlation Between the Mitophagy Expression Signature and Immune Infiltration

**Figure 4:**
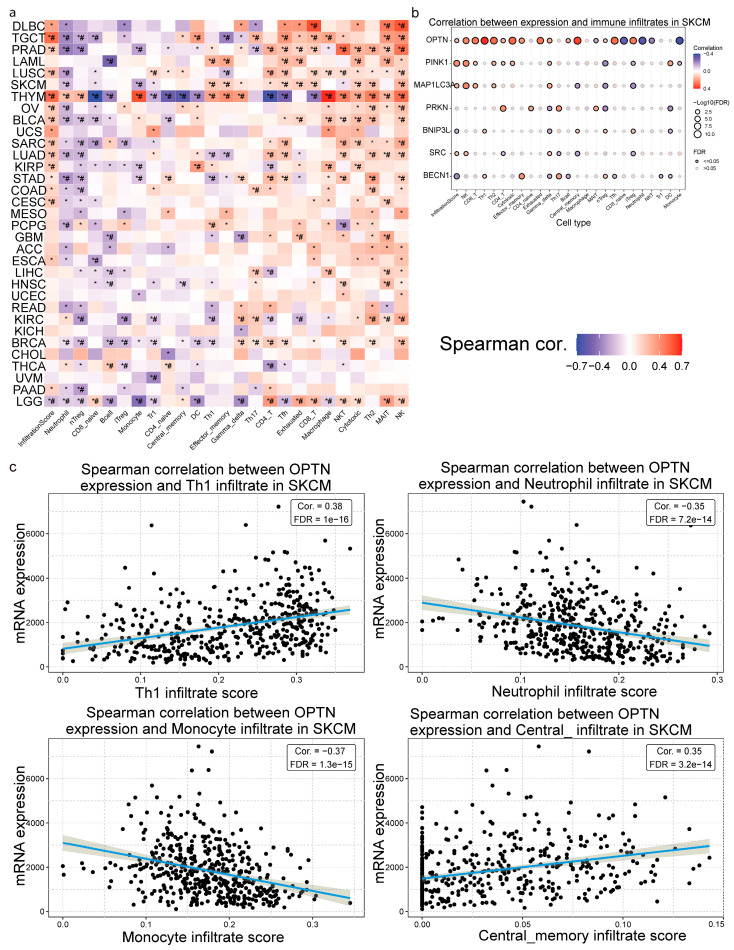
**Correlation between mitophagy-related gene expression and immune cell infiltration in pan-cancer.** (**a**) The heatmap represents the Spearman’s correlation between the infiltration of 24 immune cell types evaluated through ImmuCellAI, and the expression of the mitophagy-related gene signature across different cancer types. The color scale indicates the strength and direction of Spearman’s correlation (blue for negative, red for positive). #, Spearman’s rho <−0.4 or >0.4; *, *p* < 0.01. (**b**) Correlation between the expression of the mitophagy-related signature and immune cell infiltrates in skin melanoma (SKCM). The size of the dots represents the significance (−log_10_FDR values), while the color indicates the correlation coefficient (blue for negative, red for positive). (**c**) Scatter plots depicting the Spearman’s correlation between *OPTN* expression and specific immune cell infiltrates (Th1, neutrophils, monocytes, and central memory infiltrates) in SKCM, with trend lines and correlation coefficients. *OPTN* expression is positively correlated with the infiltration of Th1 cells and the central memory infiltrate score, and negatively correlated with the infiltration of neutrophils and monocytes in SKCM.

Next, we analyzed the mRNA expression of the mitophagy-related gene signature and its impact on 24 immune cells. Overall, the Spearman’s correlation coefficients varied across different cancer types, revealing distinct patterns of immune cell correlation with the expression of *OPTN*, *PINK1*, *MAP1LC3A*, *PRKN*, *BNIP3L*, *BECN1*, and *SRC*. Significant correlations (*p* < 0.01 and FDR < 0.01) were observed in all types of cancer. For example, diffuse large B cell lymphoma (DLBCL), tenosynovial giant cell tumors (TGCT), prostate adenocarcinomas (PRAD), and other types of tumors were positively correlated with infiltration in NK, MAIT, and CD8+ T cells, while thymomas (THYM) were negatively correlated with infiltration in CD4/CD8-naive T cells, CD4/CD8 T cells, T follicular helper (Tfh) cells, and central memory (Figure 4a). Although correlation coefficients below 0.4 indicate moderate associations, the consistent patterns observed across multiple cancer types and immune cell subsets suggest biologically meaningful relationships between mitophagy gene expression and the TME.

In addition, *PRKN*, *PINK1* and *MAP1LC3A*, expression levels were positively correlated with the infiltration score, as well as the infiltration of various immune cells, including iTreg, effector memory, exhausted, Th1, and nTreg cells in lung adenocarcinoma (LUAD).

Importantly, in skin melanoma (SKCM), *OPTN* expression was positively correlated with infiltration of Th1, Th2, central memory infiltrates, cytotoxic, CD4/CD8 T cells, NK cells, effector memory T cells, exhausted T cells, B cells, γδ T cells, Tfh and iTreg cells, while it was negatively correlated with infiltration of CD-naïve, neutrophils, NKT cells and monocytes (Figure 4b).

We also explored the correlation between the mitophagy-related signature and immune infiltration, across different tumors. For example, in breast cancer (BRCA) we found that *OPTN*, *PINK1* and *PRKN* expression was positively correlated with infiltration of NK cells, while that of *BECN1* was negatively correlated with it. In addition, *BNIP3L* and *BECN1* expression was correlated with infiltration of B cells, CD8+ T cells, dendritic cells, iTregs, cytotoxic cells, and overall immune infiltration scores. Overall, these findings highlight the diverse associations between immune infiltration and the expression of the mitophagy-related genes in pan-cancer. They also suggest that among all the mitophagy-related genes, *OPTN* seems to play a major role in modulating the immune microenvironment in SKCM, potentially influencing tumor-immune interactions and impacting melanoma progression and response to immunotherapy.

### 2.4. Correlation Between the Mitophagy Methylation Signature, Expression and Immune Infiltration

We then explored the methylation levels (beta-values, HumanMethylation450) of *MAP1LC3A*, *OPTN*, *PINK1*, *PRKN*, *SRC*, *BNIP3L*, and *BECN1* in pan-cancer and their association with gene expression and the immune cell infiltrates. Our findings indicate that the methylation levels of some of these genes differ significantly in some tumors compared to their adjacent normal tissue. For example, *OPTN* methylation (cg16907766) seems to be significantly lower in KIRP and KIRC (but not in KICH) compared to the normal kidney, but also in BLCA, CESC, PAAD, READ, SKCM, and UVM, compared to their normal adjacent tissues. On the other hand, *OPTN* exhibited hypermethylation in BRCA, CHOL, THYM, and UCEC (Figure S1). Likewise, *SRC* (cg14215077) was hypermethylated only in LGG, MESO, and UVM, and hypomethylated in CESC, ESCA, HNSC, KIRC, LIHC, LUAD, LUSC, READ, SARC, SKCM, STAD, TGCT, and USC. We also found that *PINK1* (cg10827226) was hypermethylated in KIRP and hypomethylated in TGCT. As regards *MAP1LC3A* (cg27624319), this was hypermethylated in KIRC and KIRP, but hypomethylated in ACC, COAD, KICH, LGG, MESO, PCPG, READ, SARC, SKCM, STAD, UCS, and UVM. *PRKN* (cg26245302) was also hypomethylated in most tumor types, like BLCA, KICH, MESO, OV, PCPG, READ, SKC, and THYM. *BECN1* (cg24220766) was hypomethylated in DLBCL, but hypomethylated in SKCM and UVM, while *BNIP3L* (cg13516551) did not exhibit any differentially methylated levels (Figure S1).

In addition, as anticipated, we show that the expression of most of the mitophagy-related genes was negatively correlated with their methylation pattern. For example, *OPTN* mRNA levels were significantly anti-correlated with its methylation levels in GBM (cg17405055, Spearman’s rho = −0.62, FDR = 2.14 × 10^−6^), UCEC (cg17405055, Spearman’s rho = −0.52, FDR < 0.0001), LAML (cg24176744, Spearman’s rho = −0.50, FDR < 0.0001), UVM (cg16907766, Spearman’s rho = −0.48, FDR = 7.01 × 10^−6^) and other tumors (Supplementary Table S5).

**Figure 5:**
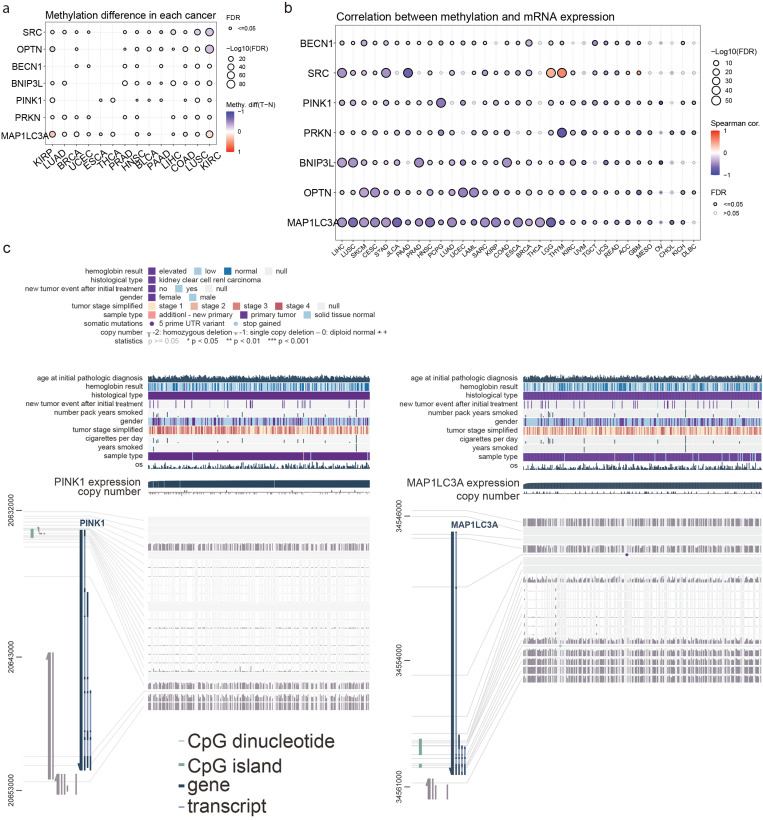
**DNA methylation analysis of mitophagy-related genes in pan-cancer and its correlation with their mRNA expression.** (**a**) The dot plot shows differential DNA methylation levels (tumor vs. normal) for mitophagy-related genes (*SRC*, *OPTN*, *PINK1*, *PRKN*, *MAP1LC3A*, *BECN1*, and *BNIP3L*) across various cancer types. The color scale represents the methylation difference (tumor–normal), and the size of the dots indicates the significance (FDR values). (**b**) The dot plot illustrates the correlation between DNA methylation levels and mRNA expression for the same set of genes across multiple cancer types. The color scale represents Spearman’s correlation coefficient, with the size of the dots indicating the significance (FDR values). (**c**) Integrative genomic analysis displaying the association between *PINK1* and *MAP1LC3A* methylation, expression and CNV, and various clinical and demographic features in KIRC. The top panel summarizes patient data [age at diagnosis, hemoglobin levels, histological type, tumor recurrence, smoking history, gender, tumor stage, sample type, and overall survival (OS)]. The middle panel shows the distribution of *PINK1* expression levels across different copy number alterations. Sections marked with ‘−2’ indicate homozygous deletions, while ‘+1’ or ‘+2’ would indicate low-level or high-level amplifications, respectively. Higher or lower expression levels of *PINK1* and *MAP1LC3A* are indicated, showing how these levels align with methylation patterns and CNVs. The bottom panel presents a detailed view of the *PINK1* (left) and *MAP1LC3A* (right) regions, showing the relationship between DNA methylation sites, CpG islands, gene structure, and expression levels. The color coding of the CpG sites (vertical lines) likely indicates their methylation status, with different shades representing varying levels of methylation.

Overall, we noted a significant hypomethylation in tumor tissues for most genes in the signature, particularly of *OPTN* and *SRC* in KIRC, indicating potential epigenetic regulation. *PRKN* and *PINK1* had similar methylation profiles with higher levels in adaptive immunity cells. *MAP1LC3A* was the only gene in the signature that exhibited hypermethylation, implying that this epigenetic process has some crucial role in cancer pathogenesis and immune cell infiltration (Figure 5a). In addition, as expected, hypermethylation seemed to generally correlate with lower gene expression, and hypomethylation with higher expression (Figure 5b).

In addition, *PRKN* methylation was inversely related to its mRNA levels in thymomas (THYM). *PRKN* exhibited higher methylation in adaptive immunity cells and lower methylation in monocytes, neutrophils, NKT, CD8-naive, and nTreg cells in LUAD, KIRC, and SKCM.

The *PINK1* methylation pattern was significantly anti-correlated with the gene’s expression in pheochromocytoma and paraganglioma tumors (PCPG), uveal melanoma (UVM), KIRC, and testicular germ cell tumors (TGCT). *PINK1* displayed higher methylation in CD4_T, nTreg, iTreg, and Tr1 cells, and lower methylation in gamma delta cells, NK, neutrophils, and Th2 cells.

An integrative genomic analysis of how DNA methylation and copy number variations within the *MAP1LC3A* gene region influence its expression in KIRC, in contrast to the *PINK1* gene region, is shown in Figure 5c. *MAP1LC3A* expression and CNVs were shown to correlate with multiple clinical and demographic parameters, whereas elevated *MAP1LC3A* expression was found to be associated with certain patient subgroups characterized by distinct clinical features, such as tumor stage, hemoglobin levels, and smoking history.

*SRC* methylation was anti-correlated with its mRNA expression in STAD, LIHC, PAAD, and LUSC, but positively correlated with it in LGG and THYM. *SRC* showed a consistent methylation pattern with *PRKN* in LUAD, KIRC, and SKCM, but had a different pattern in BRCA, with lower methylation scores observed in B cell Tfh, Th1, cytotoxic, and DC cells, and higher methylation in CD8-naive, macrophages, and Th17 cells.

*MAP1LC3A* methylation was significantly anti-correlated with its mRNA expression in most tumors. *MAP1LC3A* exhibited higher methylation in Tr, iTreg, CD8T, exhausted, cytotoxic, DC, and macrophages in KIRC, SKCM, and LUAD, and lower methylation in CD8-naive, NK, neutrophils, and Th17 cells in the same cancer types. In THCA, *MAP1LC3A* had lower methylation in neutrophils, Th2, gamma delta cells, and higher methylation in cytotoxic, DC, iTreg, and nTreg cells.

*OPTN* methylation was significantly anti-correlated with its mRNA expression in CESC, SKCM, LAML, and UCEC. *OPTN* displayed higher methylation in iTreg, Tfh, and CD4T cells in KIRC, and in CD8T, cytotoxic, central memory, and B cells in SKCM. In BRCA, *OPTN* had higher methylation in central memory and CD8-naive cells, and lower methylation in NK, NKT, and cytotoxic cells. In THCA, iTreg, nTreg, neutrophils, monocytes, and macrophages had lower methylation, while B cells showed significant upregulation (Figure 5b).

The majority of the negative correlations between the signature genes’ methylation and their mRNA levels imply that methylation of these genes is a regulatory mechanism affecting their expression in cancerous tissues.

### 2.5. Correlation Between MAP1LC3A, OPTN, SRC, PINK1, PRKN, BECN1, and BNIP3L Mutations and Immune Infiltration in Pan-Cancer

**Figure 6:**
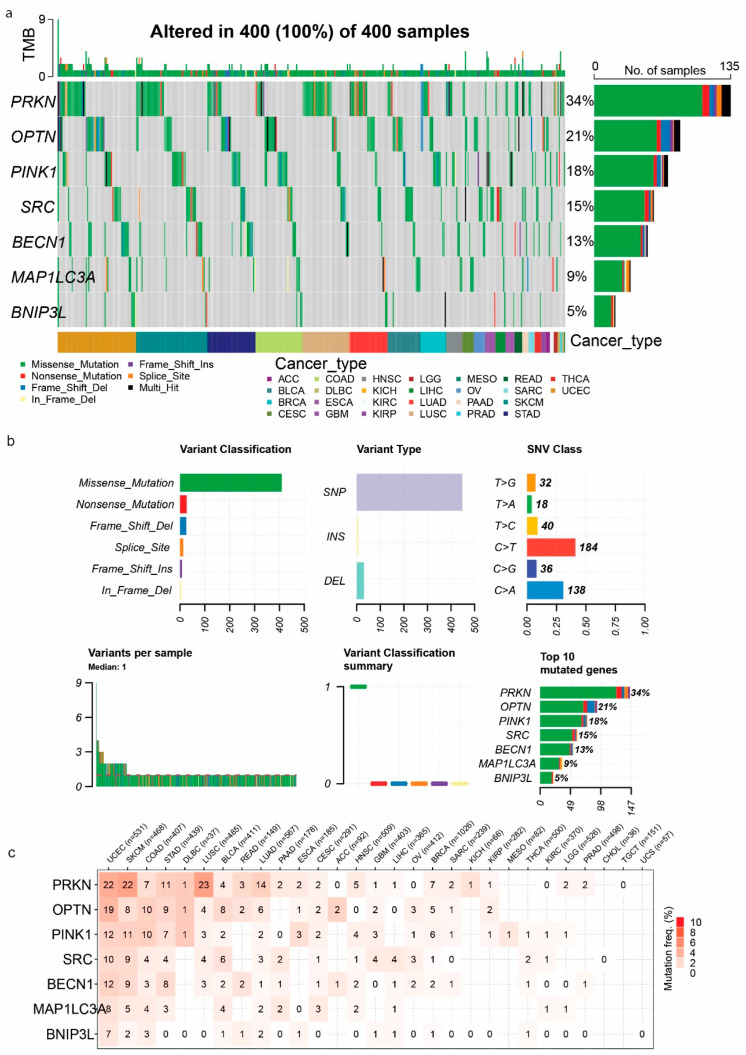
**Mutation landscape of *PRKN*, *OPTN*, *PINK1*, *SRC*, *MAP1LC3A*, *BECN1*, and *BNIP3L* across various cancer types.** (**a**) The waterfall plot presents the tumor mutation burden (TMB) per cancer sample and the mutation distribution of the mitophagy-related gene signature across 400 cancer samples in the TCGA. Each row represents a gene, and each column represents a cancer sample. Different colors indicate different types of mutations, including missense mutations, nonsense mutations, frame-shift deletions, frame-shift insertions, in-frame deletions, splice-site mutations, and multi-hit mutations. The bar plot on the right shows the percentage of samples with alterations in each gene. The bottom annotation panel indicates the cancer type for each sample. (**b**) Summary of variant classifications and types. Bar plots show the number of different mutation types (missense, nonsense, etc.) and variant types (SNP, insertion, deletion) for the five genes. The SNV class distribution is displayed, highlighting the most frequent base substitutions (184 C>T and 138 C>A). The bottom left plot shows the distribution of variants per sample, with a median of 1 variant per sample. The bottom right plot shows the variant classification summary for each gene, with the percentage of samples altered. (**c**) The heatmap shows the mutation frequency of the mitophagy-related genes across different cancer types. The numbers in each cell represent the percentage of samples with mutations in the respective gene and cancer type. The intensity of the color corresponds to the mutation frequency, with a darker red indicating a higher frequency.

The mutation landscape of the mitophagy-related genes across 400 cancer samples revealed that all samples exhibited alterations in at least one of them. We found a diverse range of mutation types, including missense mutations, being the majority, but also a lower percentage of nonsense mutations, frame-shift insertions/deletions, in-frame deletions, splice sites, and multi-hit mutations. Notably, *PRKN* exhibited the highest alteration frequency, affecting 34% of all cancer samples, followed by *OPTN* (21%), *PINK1* (18%), *SRC* (15%), *BECN1* (13%), *MAP1LC3A* (9%), and *BNIP3L* (5%) (Figure 6a). Missense mutations of C>T and C>A class were the most prevalent type of alteration across the seven genes, followed by nonsense mutations and frame-shift deletions (Figure 6b). On average, each tumor sample had 1–2 mutations (pan-cancer), while UCEC, SKCM, COAD, STAD, DLBCL, and LUSC were the most hypermutated tumors for the genes of interest (Figure 6c and Supplementary Table S6).

**Figure 7:**
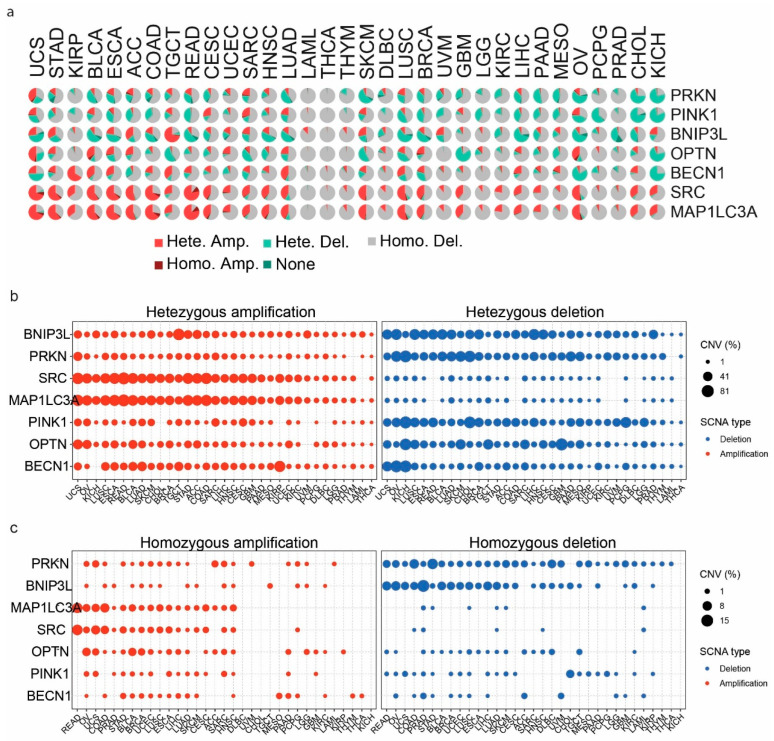
**Copy number variations in *PRKN*, *OPTN*, *PINK1*, *SRC*, *BNIP3L*, *BECN1*, and *MAP1LC3A* across different cancer types**. (**a**) The pie charts show the frequency and types of CNVs in *PRKN*, *OPTN*, *PINK1*, *SRC*, *BNIP3L*, *BECN1*, and *MAP1LC3A* across various cancer types. Each pie chart represents a cancer type, with different colors indicating the type of CNV. (**b**) The dot plots illustrate the distribution of heterozygous amplifications (left) and heterozygous deletions (right) in *PRKN*, *OPTN*, *PINK1*, *SRC*, *BNIP3L*, *BECN1*, and *MAP1LC3A* across different cancer types. The size of the dots corresponds to the percentage of samples with the respective CNV type. (**c**) The dot plots show the distribution of homozygous amplifications (left) and homozygous deletions (right) in *PRKN*, *OPTN*, *PINK1*, *SRC*, *BNIP3L*, *BECN1*, and *MAP1LC3A* across various cancer types. The size of the dots represents the percentage of samples with the respective CNV type.

The analysis of CNVs in *PRKN*, *OPTN*, *PINK1*, *SRC*, *BNIP3L*, *BECN1*, and *MAP1LC3A* across various cancer types revealed significant heterogeneity in the types and frequencies of CNVs. The highest percentages of CNVs were detected in rectal adenocarcinoma (READ), uterine carcinosarcoma (UCS), colon adenocarcinoma (COAD), glioblastoma multiforme (GBM), kidney chromophobe (KICH), and cholangiocarcinoma (CHOL). Significant heterozygous amplifications and deletions were also noted in *MAP1LC3A*, *SRC* (heterozygous amplifications), *BNIP3L*, *BECN1*, *PINK1*, and *PRKN* (heterozygous deletions) in tumors, including KIRC, DLBC, KICH, HNSC, LUSC, BRCA, LUAD, STAD, READ, and OV (Figure 7a).

We also explored the frequency of heterozygous amplifications and deletions for each gene across different cancer types. *MAP1LC3A* and *SRC* showed notable heterozygous amplifications in cancers like BRCA and LIHC. Heterozygous deletions were prevalent in *PRKN*, *BNIP3L*, *OPTN*, and *PINK1*, particularly in cancers such as LUSC, ESCA, HNSC, and COAD (Figure 7b).

Homozygous amplifications and deletions were less frequent, but still significant in certain cancers. For example, *PRKN* exhibited homozygous amplifications in cancers such as uterine carcinosarcoma (UCS) and adrenocortical carcinoma (ACC). Homozygous deletions were most prominent in *BNIP3L*, especially in prostate adenocarcinoma (PRAD), rectal adenocarcinoma (READ) and ovarian serous cystadenocarcinoma (OV) (Figure 7c).

Overall, these findings highlight the complex landscape of CNVs in *PRKN*, *OPTN*, *PINK1*, *SRC*, *BECN1*, *BNIP3L*, and *MAP1LC3A* across different cancer types, emphasizing the potential impact of these genetic alterations on cancer pathogenesis and the importance of considering CNVs in therapeutic strategies.

In addition, we noted significant correlations between CNVs and immune infiltrates, such as *PRKN* CNVs with CD4-T cells in BRCA (corr. = 0.33, *p* = 4.23 × 10^−33^, FDR = 4.11 × 10^−32^) and *PINK1* with NKT cells (corr. = 0.33, *p* = 1.18 × 10^−31^, FDR = 1.56 × 10^−30^). In KIRC, *PINK1* CNVs correlated with neutrophil infiltration (corr. = 0.44, *p* = 2.03 × 10^−29^, FDR = 2.37 × 10^−28^), and in LUSC, *SRC* CNVs correlated with infiltration of CD8-naive cells (corr. = 0.34, *p* = 1.03 × 10^−16^, FDR = 1.86 × 10^−15^). In LUAD, *PRKN* CNVs correlated with infiltration of MAIT cells (corr. = 0.41, *p* = 1.34 × 10^−24^, FDR = 4.17 × 10^−23^) and in THYM, *MAP1LC3A* CNVs correlated with gamma–delta T cell infiltration (corr. = 0.61, *p* = 9.81 × 10^−14^, FDR = 1.21 × 10^−11^). Negative correlations included *OPTN* CNVs with infiltration of CD8-naive cells in GBM (corr. = −0.44, *p* = 3.34 × 10^−8^, FDR = 1.13 × 10^−6^), *OPTN* CNVs with an infiltration of cytotoxic cells in LGG (corr. = −0.30, *p* = 3.26 × 10^−12^, FDR = 1.89 × 10^−10^), and *PINK1* CNVs with infiltration of B cells in KICH (corr. = −0.35, *p* = 4.46 × 10^−3^, FDR = 0.03). No significant negative correlations were found in BRCA for the studied genes (Supplementary Table S7).

### 2.6. Correlation Between MAP1LC3A, OPTN, SRC, BNIP3L, BECN1, PINK1, and PRKN Expression and Drug Sensitivity in Pan-Cancer

**Figure 8:**
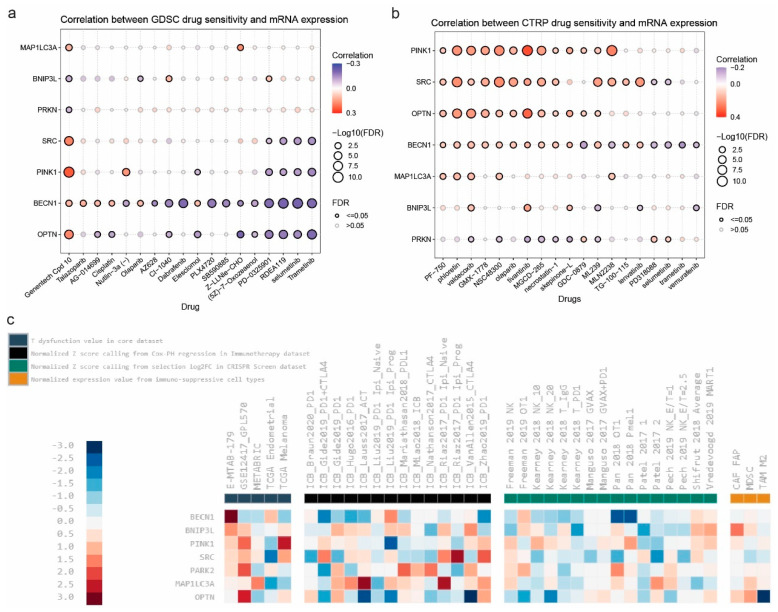
Correlation of *MAP1LC3A*, *OPTN*, *SRC*, *PINK1*, *BNIP3L*, *BECN1*, and *PRKN* expression with drug sensitivity (IC50) in pan-cancer, using the GDSC (**a**) and CTRP (**b**) drug databases. The color, from red to blue, depicts the correlation between each gene’s mRNA expression and IC50. Also, the bubble size represents the false discovery rate (FDR), with larger circles indicating stronger statistical significance. The color gradient indicates the direction and magnitude of correlation. Blue color, negative correlation; red color, positive correlation. Significant correlations (FDR < 0.05) are emphasized with bold outlines, highlighting the most critical interactions. (**c**) Regulator prioritization of the mitophagy-related gene signature. Each column is a data cohort. Genes are ranked based on their average score with multiple cohorts. The colors correspond to different score values, ranging from −3 (blue) to +3 (red).

Last, we gathered the IC50s of a wide variety of drugs among different cancer cell lines through the GDSC and CTRP databases and assessed the relationship between the mRNA values of *MAP1LC3A*, *OPTN*, *SRC*, *BNIP3L*, *BECN1*, *PINK1*, and *PRKN* and drug sensitivity. Overall, we found various significant correlations, the majority of which were positive, particularly for *PINK1*, *SRC*, *OPTN*, and *BECN1* in the CTRP, indicating that higher expression levels of these genes are associated with increased sensitivity to numerous compounds, like PF-750, phloretin, valdecoxib, GMX-1778, NSC48300, olaparib, tivantinib, MGCD-265, necrostatin-1, skepinone-L, GDC-0879, and ML239. In the GDSC drug database, *SRC*, *PINK1*, *BECN1*, and *OPTN* expression was negatively correlated with sensitivity in PD-0325901, RDEA119, selumetinib, and trametinib, while *MAP1LC3A*, *SRC*, *PINK1*, *BECN1*, and *OPTN* were positively correlated with sensitivity in Genentech Cpd 10. Likewise, *SRC* and *MAP1LC3A* mRNA levels were positively correlated with sensitivity to most of the drugs in the CTRP database. On the other hand, the mRNA levels of *PRKN* were negatively correlated with sensitivity to various drugs, including Genentech Cpd 10 (GDSC database), but also with valdecoxib, tivantinib, necrosattin-1, skepinone-L, and others (CTRP database) (Figure 8a,b). These results highlight that mitophagy-related genes play a crucial role in determining the response to various small-molecule inhibitors.

Based on the mitophagy-related signature, regulator prioritization ranked *PINK1* as the most valuable marker to follow-up on skin melanoma, *BECN1* was the most valuable marker to follow up on neuroblastoma (E-MTAB-179), as well as *PARK2*, *PINK1* and *OPTN* were the most valuable markers to follow up on acute myeloid leukemia (GSE12417_GPL570), in terms of T cell dysfunction. Similar, *MAP1LC3A* was the most valuable marker to follow up on breast cancer (METABRIC dataset). In terms of immunotherapy, *PARK2*, *BNIP3L* and *MAP1LC3A* had the highest scores in different studies using ICB. *BECN1* had negative risk scores in two CRISPR screen datasets (Pan2018 OTI and PAN2018 Pmell), while *OPTN* had a negative risk score in M2 tumor-associated macrophages (Figure 8c).

## 3. Discussion

Mitophagy plays a critical role in maintaining cellular homeostasis by removing damaged mitochondria, which is crucial for cancer cell survival under stressful conditions, such as those induced by chemotherapy [41]. Although mutations in the genes involved in this process have been linked to Parkinson’s disease [42], their involvement in different cancer types is widely unknown. By mitigating oxidative stress and preventing the accumulation of dysfunctional mitochondria, mitophagy can enhance the survival of cancer cells, thus contributing to drug resistance [29].

Here we examined a mitophagy-related signature, evaluating the patterns of differential gene expression, mutations, methylation, immune infiltration and drug sensitivity of this signature in pan-cancer. While the selected genes play established roles in mitophagy, many of them, such as *SRC* and *BECN1*, are also involved in other cellular pathways, including autophagy and cancer-related signaling. The overlap of these pathways may contribute to the observed associations with tumor progression and drug resistance. The differential expression of mitophagy-related genes across various cancer types and stages, suggests that they could serve as biomarkers for predicting patient response to treatment.

Importantly, we show that the relevance of the mitophagy-related signature varies across cancer subtypes. For example, in KIRC, the signature is strongly associated with hypomethylation and immune infiltration. In BRCA, *SRC* expression correlates with aggressive subtypes, while in SKCM, *OPTN* expression is linked with immune cell infiltration. These findings suggest that the functional impact of the signature depends on cancer subtype and molecular context, driven by differential gene expression, mutational burden, or epigenetic alterations.

Mitophagy appears to have dual roles in cancer. It can suppress tumor growth by clearing damaged mitochondria or promote survival by helping cancer cells adapt to adverse conditions, complicating therapeutic strategies. This dual role complicates the therapeutic targeting of mitophagy in cancer treatment [29].

Targeting mitophagy-related genes like *PINK1* and *PRKN* could potentially sensitize cancer cells to treatment by promoting the accumulation of dysfunctional mitochondria, thereby increasing oxidative stress and cell death. Similarly, modulating the activity of *SRC* and *OPTN* might affect autophagosome formation and selective degradation of mitochondria, influencing cancer cell survival and drug resistance [29,43,44,45]. Our analysis revealed that high *PINK1* mRNA levels in lung squamous cell carcinoma or *OPTN* in thyroid cancer are associated with improved survival. Similarly, upregulation of *MAP1LC3A* in colorectal cancer is correlated with better outcomes, reinforcing the prognostic value of these markers. Thus, combining mitophagy inhibitors with standard therapies may improve responses in aggressive cancers like these.

Decreased expression of mitophagy-related genes has been shown to cause degenerative diseases in which deficient quality control results in inflammation and the death of cell populations [2]. The *LC3* gene plays a crucial role in the formation of autophagosomes and acts as a replication platform [46,47]. The inhibition of autophagy has been shown to result in a decreased expression of *LC3*, and a decrease in autophagosomes, suggest that blocking autophagy may could be used as a potential treatment for cancer. Reducing *LC3* in triple-negative breast cancer cells significantly decreased cell viability, leading to apoptosis. On the other hand, the upregulation of *LC3* has been linked with better survival in colorectal cancer [47]. It is important to note that while *LC3A* is typically downregulated in many types of cancer, it did not show significant activation of pro-apoptotic pathways in this study, indicating that the mechanism examined by Monastyrska et al. [46] may not translate directly to humans. Additionally, we showed that *LC3A* has varying levels of dysregulation in immune infiltrates, being upregulated in innate immunity cells and downregulated in adaptive immunity cells in several cancer types.

Apart from *LC3A*, mitophagy is also interconnected with *SRC*, the high expression levels of which are linked with mitochondrial damage [48]. It has been shown that the *SRC* inhibitor PP2 effectively blocks the growth and EMT of triple-negative breast cancer cell lines [14]. Tan et al. [49] highlighted that *SRC* upregulation increases cancer aggressiveness, and *SRC* inhibitors reduce this effect. Our study supports these findings, showing upregulated SRC expression across various tumor types, indicating its role in cancer proliferation [47,49].

Alterations in *OPTN* can also affect mitophagy and mitochondrial function. Optineurin helps autophagosomes selectively consume damaged mitochondria by attaching them to them and securing them for degradation [15]. The PINK1/Parkin pathway, originally linked to Parkinson’s disease, is crucial for mitochondrial clearance. PINK1’s role in mitophagy involves its voltage-dependent translocation to the inner mitochondrial membrane and cleavage by presenilin-associated rhomboid-like protein (PARL) under normal conditions [50]. *PINK1* functions as a tumor promoter or suppressor, depending on the context [17]. *PRKN* targets damaged mitochondria for degradation, playing a crucial role in maintaining mitochondrial quality control. *PARK2* inhibits the HIPPO/YAP axis in esophageal squamous cell carcinoma, promoting YAP degradation and suppressing ESCC progression [51]. Our pan-cancer analysis revealed minimal differences between *PINK1* and *PRKN2*, suggesting that they fulfil similar functions in tumors like lung cancer. Significant changes in mRNA expression levels were identified across eight cancer types, with *PRKN* and *PINK1* being generally downregulated and SRC being upregulated. These trends indicate the role of these genes in cancer progression.

We also examined the impact of the mRNA levels of these genes on patient survival across various cancers. Most genes were associated with better survival rates when upregulated, indicating an overly activated autophagy response.

The inhibition of *SRC*, when combined with tamoxifen, has been shown to improve drug efficacy, while its upregulation increases the efficacy by inhibiting cell cycle progression. However, elevated *SRC*-3 is linked to prostate cancer recurrence and aggressive progression [52]. In our study, we show that *SRC* expression is significantly associated with overall and relapse-free survival in multiple cancers. SRC’s duality is also seen in *PINK1* and *PARK2*. Reduced *PINK1* and *PARK2* expression was noted in pRCC compared to non-neoplastic tissue, with lower protein levels documented [53]. Another study also highlighted *PINK1*’s diverse role in cancer biology [10]. In ESCC, low *PINK1* and *PARK2* levels were linked to worse differentiation, advanced stages, and poor prognosis. *PINK1* and *PARK2* were identified as independent risk factors for ESCC prognosis [54]. Our pan-cancer analysis showed an improved survival in LUSC and KIRC patients with upregulated *PINK1*, indicating their potential prognostic value. However, better survival was mostly noted when *PINK1* and *PRKN2* were downregulated, except in lung cancer. OPTN may modulate ER stress response signaling, aiding cellular homeostasis. Disruptions in *OPTN* function could contribute to pathogenesis [55]. In another study, the downregulation of HACE1 and *OPTN* proteins was observed in various cancers [56]. Our study found no clear correlation between *OPTN* expression and survival, though overexpression showed more favorable, yet not statistically significant, results. Thus, *OPTN* demonstrated a multifaceted nature.

We also examined the correlation between the specific mRNA expression of the mitophagy-related signature and pathway activity in various cancers. The dual role of *SRC* was noted in different cellular processes, showing significant activation in pathways linked to cell growth and survival, such as the RTK and TSC/mTOR pathways. These pathways are often implicated in the development of drug resistance as they promote cancer cell survival and proliferation, even in the presence of chemotherapeutic agents.

Furthermore, we examined the link between the expression of the mitophagy-related signature and immune cell infiltrates. Our findings suggest *SRC* as a prognostic biomarker in pan-cancer.

We also address the role of mutations in the mitophagy-related genes, which can further influence drug resistance. Mutations in *PRKN* and *PINK1*, for instance, may disrupt their tumor-suppressive functions, leading to an enhanced survival mechanism in cancer cells, making them more resistant to treatment.

Modulation of mitophagy-related genes has shown promise in overcoming drug resistance. For instance, inhibiting SRC activity with dasatinib sensitizes triple-negative breast cancer cells to chemotherapy by suppressing epithelial-to-mesenchymal transition [57,58]. Similarly, targeting PINK1-mediated mitophagy enhances the efficacy of cisplatin in non-small cell lung cancer by preventing the clearance of damaged mitochondria [59]. These examples illustrate how the dual role of mitophagy can be leveraged to improve treatment outcomes in specific contexts.

Importantly, we delved into the intricate relationship between genetic mutations and immune responses in cancer progression. *SRC* exhibited notable activation in pathways governing cell cycle regulation and growth signaling, while concurrently displaying inhibition in apoptosis and hormone receptor signaling pathways. We also revealed that *PRKN* displayed an intricate pattern, characterized by inhibition in cell cycle control and DNA damage response pathways, but activation in hormone receptor pathways. Similarly, *PINK1* demonstrated a dual role, inhibiting cell cycle regulation and DNA damage repair pathways, while simultaneously promoting hormone receptor signaling and RAS-MAPK pathway activity. *OPTN* displayed both activating and inhibitory effects across various pathways, including apoptosis and epithelial–mesenchymal transition.

Furthermore, we uncovered significant enrichment of immune cell infiltrates associated with *OPTN* in different cancer types, highlighting the complex regulatory mechanisms involved in cancer pathogenesis, which entail an intricate interplay between genetic mutations and immune cell interactions.

In addition, we focused on CNVs affecting the mitophagy signature across various cancers. Significant CNVs were found to affect *MAP1LC3A*, *SRC*, *PINK1*, and *PRKN* across multiple tumors, including KIRC, DLBC, KICH, HNSC, LUSC, BRCA, LUAD, STAD, READ, and OV.

Additionally, we examined how methylation in our signature associates with immune cell infiltrates across different tumors. Overall, the methylation levels of the mitophagy signature were associated with immune cell infiltrates, suggesting roles in shaping the TME. Nunes et al. [60] found that in bladder cancer, low methylation of *MAP1LC3A* could be due to compromised mitophagy mechanisms in advanced stages. These findings underscore the complex relationships between gene methylation, immune cell infiltrates and cancer development, emphasizing the role of methylation patterns in shaping the TME and influencing immune responses. Lower methylation levels were also linked with increased immune infiltrates in KIRC and BLCA, highlighting gene methylation as key determinants in tumor–immune interactions.

Future studies could explore the impact of demethylation treatments on mitophagy-related gene expression and their subsequent effects on tumor biology in specific cancer types. For instance, treatment with DNA methylation inhibitors like 5-azacytidine or decitabine could reveal whether the observed hypomethylation patterns of *PRKN* or *OPTN* in kidney renal clear cell carcinoma and other cancers are reversible and whether they influence immune cell infiltration or tumor progression.

As regards drug sensitivity, our findings provide compelling evidence for the significant role of mitophagy-related genes in influencing cancer cell sensitivity to various therapeutic compounds. The positive correlations between the expression of *PINK1*, *SRC*, *BECN1*, and *OPTN* and drug sensitivity suggest that higher levels of these genes are associated with increased vulnerability of cancer cells to treatment. This finding is particularly noteworthy because it underscores the potential of mitophagy as a therapeutic target. By enhancing the expression or activity of mitophagy-related genes, it might be possible to sensitize cancer cells to existing treatments, thereby improving their efficacy. In addition, our findings suggest that combining inhibitors of mitophagy-related pathways with traditional chemotherapies could overcome drug resistance. By inhibiting mitophagy, it might be possible to prevent cancer cells from mitigating the effects of chemotherapy, thereby enhancing treatment efficacy. Similarly, other mitophagy-related genes, including those associated with the PINK1/Parkin pathway, have been shown to facilitate drug resistance. For example, the interaction between Caveolin-1 (Cav-1) and Parkin in non-small-cell lung cancer cells bolsters resistance to cisplatin by encouraging the clearance of damaged mitochondria. Reducing Cav-1 expression diminishes this mitophagy, heightening the sensitivity of cancer cells to cisplatin. This exemplifies how the impairment of mitophagy-related gene function can weaken the cell’s capability to tolerate chemotherapy, thereby enhancing drug efficacy [61].

Mitophagy-related genes also influence resistance by affecting mitochondrial dynamics and apoptosis. For instance, a novel derivative of betulinic acid, B5G1, demonstrates anticancer properties by activating mitochondrial apoptosis and promoting mitophagy via *PINK1*. Inhibiting mitophagy with specific agents, such as mdivi-1 or bafilomycin, increases the sensitivity of drug-resistant cancer cells to B5G1, highlighting the potential of targeting mitophagy-related genes to reverse drug resistance [62,63].

While our in-silico analysis revealed significant correlations between the expression of mitophagy-related genes (e.g., *PINK1*, *SRC*, and *OPTN*) and drug sensitivity, experimental validation is critical to confirm these findings. In vitro studies, such as treating cancer cell lines with small-molecule inhibitors (e.g., SRC inhibitors like dasatinib or trametinib) or inducing mitophagy via PINK1 activators, could further elucidate the therapeutic relevance of these genes in overcoming drug resistance.

Personalized medicine offers a promising strategy to tackle cases where mitophagy contributes to cancer resistance against chemotherapy and other anti-cancer treatments. By deepening our understanding of the mechanisms that govern mitophagy and its relationship with cancer, we can devise strategies that incorporate mitophagy-related gene therapy inhibitors or inducers alongside standard chemotherapy regimens. Moreover, by identifying specific correlations between the expression of mitophagy-related genes and drug sensitivity, we can pave the way for more individualized treatment approaches. Future research should concentrate on validating these findings in clinical settings and investigating the mechanistic foundations of how mitophagy influences drug response. Additionally, exploring the interplay between mitophagy and other cellular pathways could yield valuable insights into combination therapies that leverage these vulnerabilities. Overall, this approach emphasizes the significance of integrating mitophagy-related biomarkers into cancer treatment strategies to improve therapeutic outcomes.

## 4. Materials and Methods

### 4.1. Gene Selection and Differential Expression

We selected genes with well-established roles in mitophagy, as evidenced by prior literature and pathway annotations in the Kyoto Encyclopedia of Genes and Genomes (KEGG) database (accessed on 1 July 2024). Priority was given to genes with documented involvement in cancer progression, mitochondrial quality control, and autophagy. The final signature included *PRKN*, *PINK1*, *MAP1LC3A*, *SRC*, *BNIP3L*, *BECN1* and *OPTN*, which represent a core set of mitophagy regulators.

We extracted normalized and batch-correlated RSEM mRNA levels (level 3) from the Cancer Genome Atlas (TCGA) [64] through the GDC Data Portal (Data Release 41.0—28 August 2024) to assess the differential expression of *OPTN*, *MAP1LC3B*, *SRC*, *BNIP3L*, *BECN1*, *PINK1*, and *PRKN* across 12 cancer types and their paired normal tissues.

We also assessed the patient’s overall (OS), progression-free (PFS), disease-specific survival (DSS), and disease-free interval (DFI) based on the mitophagy-related gene expression, using *survival* in R (v. 4.1.1), the Cox proportional hazards model and log-rank tests.

In addition, we explored mitophagy-related gene expression across tumors of different stages. To this end, we used 4 types of stage (pathologic, clinical, masaoka (for THYM only), and igcccg stage (for TGCT only)).

### 4.2. Pathway Activity

We estimated the difference in mitophagy-related gene expression between pathway activity groups (activation and inhibition). To this end, we used reverse phase protein array (RPPA) data from the Cancer Proteome Atlas (TCPA) [65] to calculate the pathway activity scores of 10 cancer-related pathways in pan-cancer, as previously described in detail [66,67,68].

### 4.3. Immune Infiltration

We estimated the association between the mitophagy-related signature’s mRNA levels and the infiltration of immune cells in the tumor, using Spearman’s correlation. The infiltrates of 24 immune cells were evaluated using ImmuCellAI [69].

### 4.4. Mutations

Single nucleotide variations (SNVs) and copy number variations (CNVs) affecting the mitophagy-related signature genes were extracted from the TCGA. High-confidence mutations were selected based on functional impact scores annotated using the ANNOVAR tool, with a focus on missense, nonsense, and frameshift mutations. CNV data were processed through GISTIC2.0 [70].

### 4.5. Differential Methylation

Illumina Human Methylation 450 k (level 3) data were downloaded from the TCGA. As there are multiple methylation sites within each gene, we used multiple tags for each genomic site and filtered out the sites being most negatively correlated with gene expression prior to the analysis.

MEXPRESS was also used to visualize DNA methylation, expression and clinical data [71]. DNA methylation values (beta values, ranging between 0 and 1, represent the ratio of the intensity of the methylated bead type to the combined locus intensity) were recorded for each array probe in each sample via BeadStudio 2.1. software. Microarray probes were mapped onto the human genome coordinates using Xena probeMap derived from the GEO GPL13534 record.

### 4.6. Drug Sensitivity

We collected the inhibitory concentrations (IC50s) of various molecules across different cell lines and the corresponding mRNA levels from the Genomics of Drug Sensitivity in Cancer (GDSC v2) [72] and the Genomics of Therapeutics Response Portal (CTRP v2) [73]. The CTRP v2 provides information on 481 small-molecule probes and drugs that selectively target distinct nodes in cell circuitry and that collectively modulate a broad array of cell processes. In addition, GDSC v2 contains 969 cell lines, 297 compounds, and 243,466 IC50s (assay: CellTitreGlo; duration: 72 h). We correlated the gene expression patterns and drug IC50s using the Pearson’s correlation coefficient with threshold of |r| > 0.3 and a false discovery rate (FDR) < 0.05. Sensitivity was classified as positive if higher gene expression correlated with lower IC50 values (greater sensitivity) and negative if the correlation was inverse. Cross-validation was performed using randomly stratified subsets of the data, and key findings were replicated across both the GDSC and CTRP databases to ensure robustness.

We also used the Tumor Immune Dysfunction and Exclusion (TIDE) algorithm [74,75] (http://tide.dfci.harvard.edu/, accessed on 3 August 2024) to find associations between the mRNA expression of the mitophagy-related signature and immune checkpoint inhibition therapy outcomes, through regular prioritization. To this end, we ranked our gene set based on dysfunction and risk scores computed from different clinical studies and CRISPR screens. Z-transformation was applied to unify the scores and the mean score was subtracted divided by the standard deviation of all values.

### 4.7. Selection and Description of Participants

Level 3 mRNA expression data were extracted from 9478 tumor samples and their adjacent normal tissue across 27 cancer types. Normalized mRNA expression data (level 3 RSEM) were obtained from TCGA and subsequently processed for batch effects using the Combat algorithm implemented in the ‘*sva*’ R package (v. 4.1.1). This method adjusts for potential confounding factors across datasets, ensuring comparability between samples [76]. For immune infiltration, we analysed 4950 samples from 33 cancer types. SNVs affecting the gene signature were extracted from 10,234 samples from 33 cancer types.

### 4.8. Statistics

For the analysis of differential gene expression and methylation between cancer and normal tissues, the *p*-values were estimated using a *t*-test and then adjusted by FDR. FDR ≤ 0.05 was considered statistically significant. To assess differences in gene expression across tumor stages, we used the Mann–Kendall Trend test. The difference in pathway activity score between subgroups was also defined by Student’s *t*-test, and *p*-values were FDR-adjusted. Mitophagy-related gene expression was correlated with mutations and methylation, using Spearman’s test. Pearson’s test was used to correlate mRNA expression with drug IC50. All *p*-values were adjusted by FDR. Raw data and bioinformatics scripts used in this study are available in Figshare (10.6084/m9.figshare.28079306) upon publication to ensure reproducibility.

## 5. Conclusions

Our findings indicate that the relevance of mitophagy varies across cancer subtypes and their molecular characteristics. The expression of this signature impacts different aspects of cancer progression, such as drug sensitivity, immune cell infiltration and the activity of different cancer-related pathways. By understanding the mechanisms through which mitophagy-related genes contribute to drug tolerance, new therapeutic strategies can be developed to enhance the sensitivity of cancer cells to treatment. Here, we support that the comprehensive analysis of gene expression, pathway activity, methylation and immune infiltration provides a robust framework for exploring these mechanisms and their implications in personalized cancer therapy.

## Data Availability

Genomic data were extracted from TCGA (https://portal.gdc.cancer.gov/, accessed on 1 May 2024). Small molecule drugs’ data were extracted from GDSC (https://www.cancerrxgene.org/, accessed on 1 May 2024) and CTRP (https://portals.broadinstitute.org/ctrp/, accessed on 1 May 2024). Immunogenomic data analysis was performed using ImmuCellAI (http://bioinfo.life.hust.edu.cn/ImmuCellAI#!/, accessed on 1 May 2024).

## Supplementary Materials

The following supporting information can be downloaded at: https://www.mdpi.com/article/10.3390/ijms26020448/s1.

## Abbreviations

ALS	amyotrophic lateral sclerosis
AR	androgen receptor
BRCA	breast cancer
CDR	cancer drug resistance
CNV	copy number variation
COAD	colon adenocarcinoma
CTRP	cancer therapeutics response portal
DC	dendritic cells
DFI	disease-free interval
DLBC	diffuse large B-cell lymphoma
EMT	epithelial–mesenchymal transition
ER	estrogen receptor
FDR	false discovery rate
GBM	glioblastoma multiforme
GDSC	genomics of drug sensitivity in cancer
HNSC	head and neck squamous cell carcinoma
ICB	immune checkpoint blockade
IC50	half maximal inhibitory concentration
KICH	kidney chromophobe
KIRC	kidney renal clear cell carcinoma
LGG	lower grade glioma
LIHC	liver hepatocellular carcinoma
LUAD	lung adenocarcinoma
LUSC	lung squamous cell carcinoma
MAP1LC3A	microtubule-associated protein 1 light chain 3 alpha
NK	natural killer (cells)
NKT	natural killer T (cells)
OPTN	optineurin
OS	overall survival
OV	ovarian cancer
PCPG	pheochromocytoma and paraganglioma
PFS	progression-free survival
PINK1	PTEN-induced putative kinase 1
PRAD	prostate adenocarcinoma
PRKN	Parkin RBR e3 ubiquitin protein ligase
PTM	post-translational modification
RTK	receptor tyrosine kinase
SKCM	skin cutaneous melanoma
SRC	SRC proto-oncogene, non-receptor tyrosine kinase
STAD	stomach adenocarcinoma
TCPA	The Cancer Proteome Atlas
TCGA	The Cancer Genome Atlas
TGCT	testicular germ cell tumors
THCA	thyroid carcinoma
THYM	thymoma
TIDE	tumor immune dysfunction and exclusion
TME	tumor microenvironment
TSC/mTOR	tuberous sclerosis complex / mechanistic target of rapamycin
UCEC	uterine corpus endometrial carcinoma
