# Supplementary material for: A Multi-Omics Analysis of a Mitophagy-Related Signature in Pan-Cancer
Source: Int J Mol Sci. 2025 Jan 7;26(2):448. doi: 10.3390/ijms26020448 (PMC11765132; doi:10.3390/ijms26020448)
Supplement: Supplementary file 1 [file ijms-26-00448-s001.zip › ijms-3397756-supplementary figure-submit 2.pdf]

International Journal of
Molecular Sciences

MDPI

Supplementary Materials

A multi-omics analysis of a mitophagy-related signature in pan-cancer

Nora Agir [1,2], Ilias Georgakopoulos-Soares [3] and Apostolos Zaravinos [1,2,*]

[1] Department of Life Sciences, School of Sciences, European University Cyprus, Nicosia, Cyprus; na202005@students.euc.ac.cy
[2] Cancer Genetics, Genomics and Systems Biology Laboratory, Basic and Translational Cancer Research Center (BTCRC), Nicosia, Cyprus; a.zaravinos@euc.ac.cy
[3] Institute for Personalized Medicine, Department of Biochemistry and Molecular Biology, The Pennsylvania State University College of Medicine, Hershey, PA, USA; izg5139@psu.edu
* Correspondence: a.zaravinos@euc.ac.cy

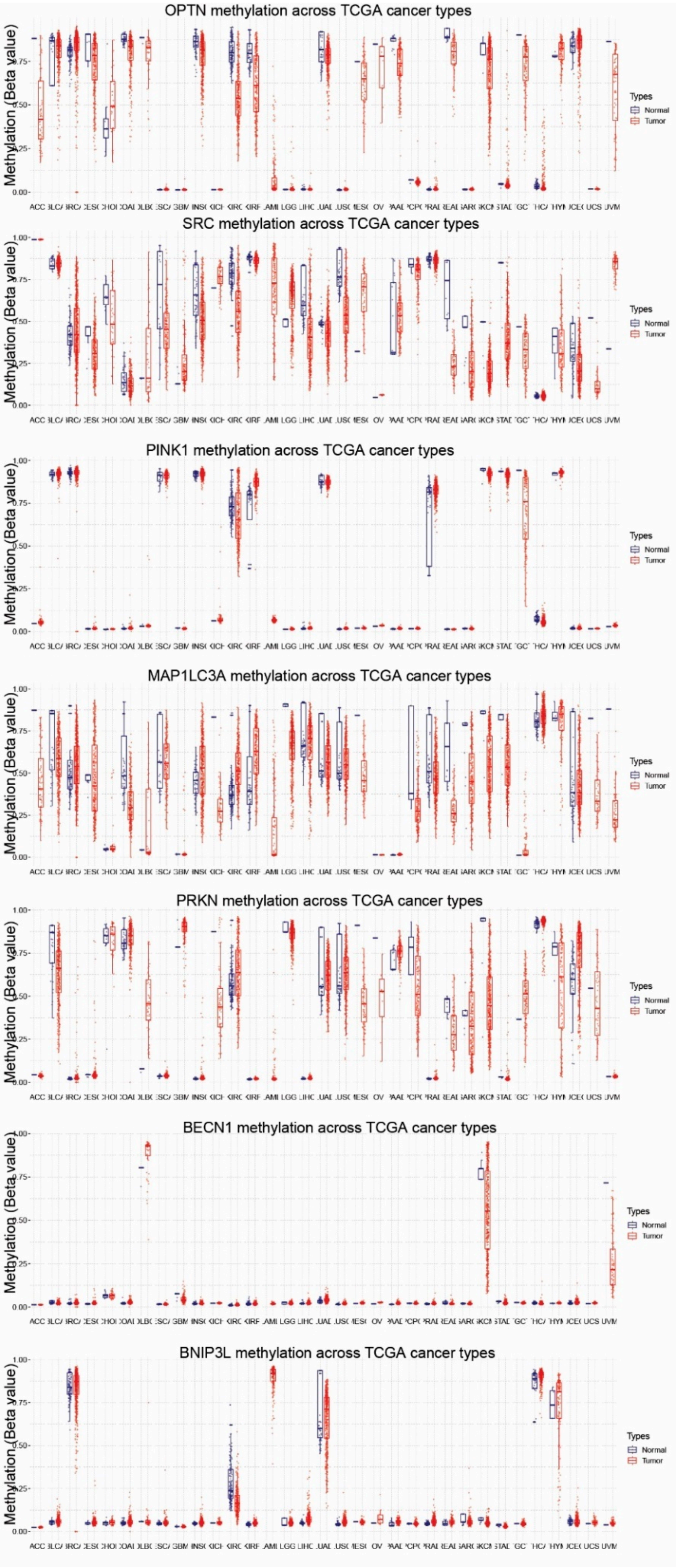

Figure S1. Differential methylation of the mitophagy-related gene signature across different cancer types compared to the adjacent normal tissue. Methylation values (beta values,

HumanMethylation450) of the genes *MAP1LC3A, OPTN, PINK1, PRKN, SRC, BNIP3L* and *BECN1* exhibited significant differences in some tumors.

Suppl. Table S1. Differential expression of the mitophagy signature in pan-cancer.
Suppl. Table S2. Differential expression of the mitophagy signature in molecular subtypes of different cancers.
Suppl. Table S3. Differential expression of the mitophagy signature in different cancer stages.
Suppl. Table S4. Differential expression of the mitophagy signature and patient outcomes across different cancers.
Suppl. Table S5. Spearman's correlation between the mitophagy-related signature and methylation patterns.
Suppl. Table S6. Mutations of the mitophagy-related signature in pan-cancer.
Suppl. Table S7. Spearman's correlation between CNVs affecting the mitophagy signature and immune infiltrates in pan-cancer.
